# High Co-infection Status of Novel Porcine Parvovirus 7 With Porcine Circovirus 3 in Sows That Experienced Reproductive Failure

**DOI:** 10.3389/fvets.2021.695553

**Published:** 2021-07-29

**Authors:** Jinhui Mai, Dongliang Wang, Yawen Zou, Sujiao Zhang, Chenguang Meng, Aibing Wang, Naidong Wang

**Affiliations:** Hunan Provincial Key Laboratory of Protein Engineering in Animal Vaccines, Laboratory of Functional Proteomics (LFP), Research Center of Reverse Vaccinology (RCRV), College of Veterinary Medicine, Hunan Agricultural University, Changsha, China

**Keywords:** porcine parvovirus 7, porcine circovirus type 3, co-infections, viremia, RT-PCR

## Abstract

Porcine parvoviruses (PPVs) and porcine circoviruses (PCVs) infect pigs worldwide, with PPV1–7 and PCV2 infections common in pigs. Although PPV7 was only identified in 2016, co-infection of PPV7 and PCV2 is already common, and PPV7 may stimulate PCV2 replication. PCV3, a novel type of circovirus, is prevalent in pig populations worldwide and considered to cause reproductive disorders and dermatitis nephrotic syndrome. In recent studies, pigs were commonly infected with both PCV3 and PPV7. Our objective was to investigate the co-infections between PPV7 and PCV3 in samples from swine on farms in Hunan, China, and assess the potential impacts of PPV7 on PCV3 viremia. A total of 209 samples, known to be positive (105) or negative (104) for PCV3, were randomly selected from serum samples that were collected from commercial swine herds in seven regions from 2016 to 2018 in our previous studies; these samples were subjected to real-time PCR to detect PPV7. Of these samples, 23% (48/209) were positive for PPV7. Furthermore, the PPV7 positive rate was significantly higher in PCV3 positive serum (31.4%, 33/105) than in PCV3 negative serum (14.4%, 15/104). Another 62 PCV3 positive sow serum samples and 20 PCV3 positive aborted fetuses were selected from 2015 to 2016 in our other previous study. These samples were designated as being from farms with or without long-standing histories of reproductive failure (RF or non-RF), respectively, and they were also subjected to real-time PCR to detect PPV7 and to determine whether PPV7 affected PCV3 viremia. Among the 62 serum samples (39 PCV3 positive RF-serum and 23 PCV3 positive non-RF-serum), 45.1% (28/62) were positive for PPV7 and PCV3, and the PPV7 positive rate was significantly higher in PCV3 positive RF-serum (51.2%, 20/39) than in PCV3 positive non-RF-serum (34.8%, 8/23). In addition, there was a higher positive rate of PPV7 (55%, 11/20) in PCV3 positive aborted fetus samples. In addition, the copy number of PCV3 in PPV7 positive samples was significantly higher than that in PPV7 negative serum samples. Based on these findings, we concluded that PPV7 may stimulate PCV3 replication.

## Introduction

Porcine parvoviruses (PPVs) have been prevalent in pigs globally, and PPV1 is considered as one of the main pathogens causing reproductive failure in pigs around the world (1). However, genotypes PPV2–PPV6 with pathogenic potential were also detected, e.g., by genome sequencing. Porcine parvovirus 7 (PPV7) was initially identified in 2016 by metagenomics sequencing of rectal swabs from healthy adult pigs in the United States and subsequently from pigs in Brazil, China, South Korea, Poland, and Sweden. In China, PPV7 is already prevalent in Guangxi, Hunan, Anhui, Fujian, Shandong, and Northeast China (2, 3), although the detailed information of its pathogenicity in pigs remains unavailable. Regarding novel PPVs, PPV4, PPV6, and PPV7 were detected in aborted fetuses, which implied that these viruses may cause reproductive failure (4–6). Moreover, detection of PPV7 in semen implies that this virus may cause reproductive dysfunction through vertical transmission (7).

PPV7 is a single-stranded DNA (ssDNA, ~4 kb), non-enveloped virus, with low homology with PPV1–6 (~30%). It belongs to the family *Parvovirinae* and is an emerging species of the genus *Chapparvovirus*. PPV7 can be isolated from healthy and sick pigs of all ages and was present in various tissues (liver, lung, lymph node, kidney, and spleen). Nucleotide mutation rates of *NS1* and *cap* genes of PPV7 were higher than those of PPV1–4 (8), perhaps enabling PPV7 to adapt to various environmental conditions and posing a major threat to health security of pig herds.

PPV1–7 and porcine circovirus 2 (PCV2) co-infections are common in pigs. In recent studies, the level of PCV2 viremia was greater in serum samples that were positive for PPV1 and PPV7 than in those that were negative for PPVs (9, 10). Furthermore, there was a correlation between the Ct values of PPV7 and PCV2 (11). As a consequence, we inferred that, in addition to PPV1, PPV7 may potentially act as a co-factor infection by stimulating the replication of PCV2. PCV3, a novel type of circovirus discovered in 2016, is prevalent in many countries around the world and is regarded as causing reproductive disorders and dermatitis nephrotic syndrome, although the pathogenesis is not well established. It was reported that PCV3 positive samples have a high co-infection rate with PPV7 (12), although nothing is known about the impact of PPV7 on PCV3 viremia. In this study, we investigated co-infections between PPV7 and PCV3 in samples from swine on farms in Hunan, China, and assessed potential impacts of PPV7 on PCV3 viremia.

## Materials and Methods

### Serum and Aborted Fetuses

We previously detected PCV3 IgG antibodies in sow sera from commercial swine herds (*n* = 1038) in seven regions of Hunan Province, China using capsid protein-based indirect ELISA (13). Among them, a total of 209 serum samples (105 PCV3 positive and 104 PCV3 negative serum samples, Table 1), based on PCV3 detection by quantitative PCR (qPCR), as described (13, 14), were randomly selected and used to determine PPV7 prevalence in the present study. In other studies, we reported identification of PCV3 (using qPCR and ELISA, respectively) in sow sera (*n* = 190), which were selected from the farms (A–E) with or without reproductive failure (RF) in various regions in Hunan, China (14). In more detail (Table 2), 85 samples (with 39 PCV3 positive) were from sows that had aborted or had a history of reproductive failure (+RF), whereas the remaining 105 (with 23 PCV3 positive) were from healthy sows (from herds with no history of reproductive failure, –RF), among which copy numbers of PCV3 genome based on qPCR were determined and reported (13, 14). It was noteworthy that the PCV3 positive rate was significantly higher in sows with reproductive failure [+RF, 45.9% (39/85)] than in healthy sows [–RF, 21.9% (23/105)] (14). In addition, 60.6% (20/33) of aborted fetuses from Farms C and E were positive for PCV3 (13), based on qPCR assays (Table 2).

**Table 1:** Presence of PPV7 in PCV3 positive and negative serum samples.

**Region**	**PCV3 positive**	**PPV7 positive**	**PCV3 negative**	**PPV7 negative**
Chenzhou	15	2	15	2
Hengyang	15	9	15	3
Shaoyang	15	7	15	4
Yueyang	15	4	14	1
Changde	15	4	15	2
Yiyang	15	5	15	3
Loudi	15	2	15	0
Total	105	33	104	15

**Table 2:** Presence of PCV3 and PPV7 co-infections in serum of sows, with and without reproductive failure (RF), and in aborted fetuses.

**Farm**	**No**.	**PCV3 positive**				**Co-infection with PPV7**			
		**Sow serum**		**Aborted fetus**		**Sow serum**		**Aborted fetus**	
		**+RF**	**–RF**			**+RF**	**–RF**		
A	23	3/8	2/15	–	–	3/3	1/2	–	–
B	24	3/9	2/15	–	–	2/3	1/2	–	–
C	41	11/26	6/15	17	11/17	5/11	1/6	11	6/11
D	22	2/7	3/15	–	–	2/2	2/3	–	–
E	35	13/20	7/15	16	9/16	5/13	2/7	9	5/9
F	20	3/5	2/15	–	–	1/3	0/2	–	–
G	25	4/10	1/15	–	–	2/4	0/1	–	–
Total	190	39/85(45.9%)	23/105 (21.9%)	33	20/33 (60.6%)	20/39(51.2%)	8/23 (34.8%)	20	11/20 (55%)

As these important samples have already been tested for PCV3 and its viral load, they can also be used to detect co-infection with PPV7, facilitating an in-depth study of the co-infection of PCV3 and PPV7 and the interaction by co-infection to enhance or stimulate virus replication.

### Real-Time PCR Assay for PCV3 and PPV7

qPCR for copy numbers of PCV3 genomic DNA with primers (QP3-F: YAGTGCTCCCCATTGAACGG and QP3-R: GCTCCAAGACGACCCTTATGC) in our previous report (13) was used to determine the copy number of PCV3 in the samples. In addition, a SYBR Green real-time PCR assay with primers (F1: GCGACCAGTCGAAAGTCTTC and R1: TTGGTGTTGCCCATTCTGTA) targeting a 165-bp region of PPV7, the conserved capsid gene for PPV7 detection, was done, as described (15). Based on results of real-time PCR, samples were deemed negative or positive for PCV3 and for PPV7.

In brief, we used a 20-μl reaction mixture containing 10 μl of AceQ qPCR SYBR Green Master Mix (Vazyme Biotech Co., Piscataway, NJ, USA), 0.4 μl PCV3 primer pairs or 0.6 μl PPV7 primer pairs (10 μM), 0.4 μl of 50 × ROX Reference Dye 1, 2 μl of DNA template, and 6.8 μl of RNase-free ddH_2_O. In addition, a pSP72 plasmid clone containing the full-length *cap* gene of PCV3 (pSP72-PCV3; GenBank accession number KY484769) or the full-length *VP2* gene of PPV7 (pSP72-PPV7; GenBank accession number KU563733) and ddH_2_O were used as positive and negative controls, respectively. Copy numbers of viral genomic DNA extracted from samples were calculated based on a standard curve.

### Statistical Analyses

All statistical analyses were performed using SPSS 21.0 software (SPSS Inc., Chicago, IL, USA) and GraphPad Prism version 8.0.0 for Windows (GraphPad Software, San Diego, CA, USA; www.graphpad.com). PCV3 and PPV7 serum categories were investigated using Fisher's exact test by pairwise comparisons. The one-way ANOVA was used for statistical comparison between PCV3 and PPV7 serum categories expressed as copy numbers. Pearson's correlations of copy numbers in PCV3 and PPV7 positive samples were determined. Statistical significance was set at *p* < 0.05, and confidence intervals were calculated.

## Results

### PPV7 Infections Occur Frequently in Pigs Affected With PCV3

The 209 samples (105 PCV3 positive and 104 PCV3 negative serum samples), derived from our previous study (13), were randomly selected from each region in Hunan, China, from 2016 to 2018. Among these 209 serum samples, 23% (48/209) were positive for PPV7. Of these, 31.4% (33/105) were positive for PPV7 in PCV3 positive serum samples (Table 1), whereas PPV7 was detected in 14.4% (15/104) of the randomly selected PCV3 negative samples (Table 1). The PPV7 positive rate was significantly higher (2.2 times) in PCV3 positive serum samples (31.4%) than in PCV3 negative serum samples (14.4%).

In this study, we also used sow sera and aborted fetuses that had been collected between 2015 and 2016 from seven sow farms with histories of long-standing reproductive problems (14). Among the 190 serum samples, there were 62 PCV3 positive and 128 PCV3 negative (14), whereas 24.7% (47/190) were positive for PPV7 (Table 2). The PPV7 positive rate was significantly higher (3.0 times) in PCV3 positive serum samples (28/62, 45.1%) than in PCV3 negative serum samples (19/128,14.8%).

The PPV7 detection rates in PCV3 positive serum samples with RF (+RF) were 51.2% (20/39), whereas they were only 34.8% (8/23) in PCV3 positive sera without RF (–RF). Furthermore, among 33 aborted fetuses from Farms C and E that had 20 PCV3 positive fetuses (14), 55% (11/20) were positive for both PPV7 and PCV3 (Table 2). In summary, the PPV7 positive rate was 1.5 times higher in PCV3 positive serum from sows with RF (+RF) vs. without RF (–RF); furthermore, there was a higher PPV7 prevalence (55%) in aborted fetus samples.

### PCV3 Viremia Is Higher in PPV7 Positive Pigs

To evaluate impacts of PCV3 and PPV7 co-infections on their viremia, 190 sow serum samples (+RF and –RF) used in a previous report (14) were divided into the following groups: PCV3–PPV7 positive (*n* = 28), PCV3 positive–PPV7 negative (*n* = 34), and PCV3 negative–PPV7 positive (*n* = 19).

**Figure 1:**
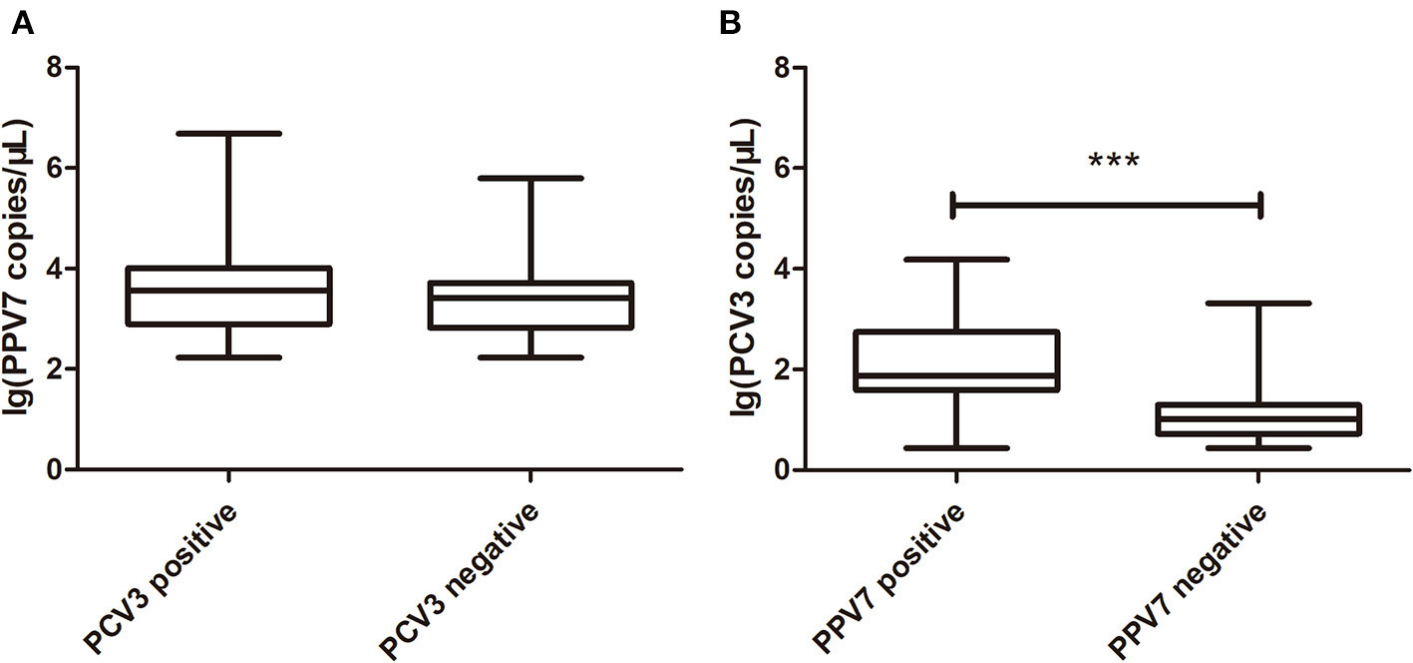
Boxplot comparison of real-time PCR copy number of PPV7 and PCV3. **(A)** Boxplot comparison of real-time PCR copy number of PPV7 in PCV3 positive (*n* = 28) and PCV3 negative (*n* = 19) serum samples. **(B)** Boxplot comparison of real-time PCR copy number of PCV3 in PPV7 positive (*n* = 28) and PPV7 negative (*n* = 27) serum samples. *** *p* < 0.001.

**Figure 2:**
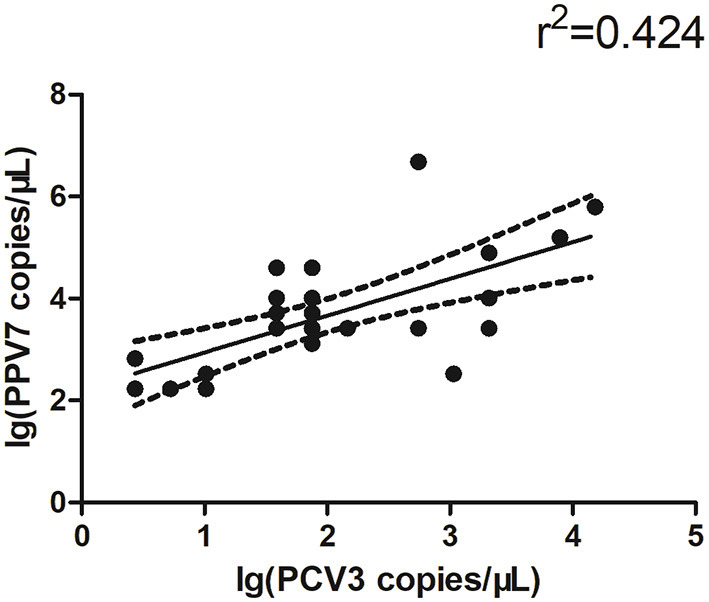
Scatterplots with trends for real-time PCR copy number for PPV7 and PCV3 positive samples (*n* = 28, *p* = 0.0002).

The copy number of PPV7 in PCV3 positive and negative serum samples was detected by real-time PCR; there was no significant difference in PPV7 between PCV3 positive (*n* = 28) and negative (*n* = 19) samples (Figure 1A). However, the copy number of PCV3 in PPV7 positive samples (*n* = 28, PCV3–PPV7 positive groups) was higher (*p* < 0.001) than that in PPV7 negative serum samples (*n* = 27, selected from 34 samples of PCV3 positive–PPV7 negative groups) (Figure 1B), and there was a very high correlation (*p* = 0.0002) in copy number between PCV3 and PPV7 from PCV3–PPV7 positive group samples (Figure 2). The linear correlation coefficient (r) between PPV7 and PCV3 copy numbers was 0.651. As the square of correlation (r^2^) score was 0.424, 42.4% of PCV3 copy number could be accounted for by PPV7 copy number.

## Discussion

PPV1 co-infects with PCV2 and PCV3, porcine reproductive and respiratory syndrome virus (PRRSV), pseudorabies virus (PRV), and classical swine fever virus (CSFV). The prevalence of PPV1–PCV2 co-infections is high, and PPV1 may trigger PCV2 associated disease (PCVAD) by supporting PCV2 replication, and increase PCVAD severity (e.g., pathological lesions in lymphoid tissues) (16). In addition, there are co-infections between novel PPVs and other well-known pathogens (e.g., PPVs, PCV2, PCV3, PRRSV, and TTSU) (3). Infections with PPV7 may become chronic, and PPV7 may contribute to virus persistence, with continuous excretion of virus in feces (17). In addition, fattening pigs without clinical symptoms had a high viral load (Ct <25), for PPV7 in their feces; therefore, variations in PPV7 viral loads may indicate various effects of PPV7 infection in pigs (17), or perhaps other conditions (e.g., co-infection) that made PPV7 pathogenic in pigs.

The prevalence of PPV7 ranged from 8.6 to 61.5% (2, 9, 11, 17–20). In our study of pigs from Hunan, China, the prevalence of PPV7 for both PCV3 negative and positive serum samples combined was 23% (48/209), and the prevalence in sow serum samples with or without RF was 24.7% (47/190). There was no basis to conclude that PPV7 contributed to all observed pathologic changes, as not all pathogens were consistently detected in diseased pigs and the prevalence of PPV7 in serum samples was higher than that in other tissues (18). Furthermore, none of the diseased pigs was only infected with PPV7. Therefore, it remains to be determined whether infection with PPV7 *per se* induces disease in pigs. It was also reported that the positive rate of PPV7 in PCV2 positive pig farms was significantly higher than that in negative farms (65.5 vs. 5.7%, respectively) (18). Moreover, the co-infection rate of PPV7 and PCV2 was high (17.4–59.5%) composed of 17.4% (67/385) and 59.5% (147/247) in Guangxi, 18.2% (29/159) in Poland, and 17.5% (21/120) in Anhui, respectively (9, 11, 19, 21). Therefore, it was speculated that PPV7 was an important cofactor of PCVAD (9). Although clinical symptoms and pathology of PPV7 remain unclear, it may act as a co-factor of disease caused by other porcine pathogens, or it may trigger disease development.

The co-infection rate of PCV3 and PPV7 was 9.1% (11/120) in samples from commercial farms with various clinical symptoms, including respiratory and gastrointestinal (19). In another report, in PCV3 positive samples, PCV3 had a high co-infection rate with both PPV6 (60.0%, 21/35) and PPV7 (74.3%, 26/35) (12). Based on these data, we inferred that there is a possible association between PCV3 and PPV7 infections. In our study, PCV3 also had a high co-infection rate with PPV7 [45.1% (28/62), 55% (11/20)]. Since both circovirus and parvovirus are ssDNA viruses, active proliferation of target cells is required for efficient viral replication. Virus-induced lymphocyte proliferation or immunosuppression can enhance the susceptibility to other virus replication and infection (22–25). For PCV2, its infection directly targets immune cells and causes immunosuppression (26–28), which leads to secondary or mixed infections with other pathogens. Furthermore, evidence that PPV-induced immune dysfunction could promote PCV2 replication (29) supports our notion that a co-infection of PPV7 and PCV3 could enhance the pathogenicity of the latter virus.

In this study, the PPV7 positive rate was statistically significantly higher in PCV3 positive versus PCV3 negative samples, suggesting that PCV3 may also cause immunosuppression, similar to PCV2, leading to secondary infection. Interestingly, co-infection with PPV7 and PCV3 in sow serum with RF (+RF) was significantly higher than that in sow serum without RF (–RF), and we also noted a higher PPV7 prevalence in aborted fetus samples. Furthermore, there were higher PCV3 viral loads in samples that were PPV7 positive compared with PPV7 negative. It has been suggested that PPV7 may stimulate the replication of PCV2 (11, 30). We speculated that PPV7 stimulated the replication of PCV3, thereby enhancing PCV3 viremia. Based on the present results and previous studies, we concluded that PPV7 may be an important co-factor triggering PCV2 and PCV3-associated diseases. Regardless, the pathogenesis of PPV7 infections, with or without PCV3 co-infection, needs to be further confirmed. More frequent multifactorial co-infection in clinical conditions contributes to a range of disease syndromes and is one of the most difficult problems in swine production, where next-generation sequencing (NGS) will gain a new insight into how co-factor infections interact to cause syndromes.

## Data Availability Statement

The original contributions presented in the study are included in the article/supplementary material, further inquiries can be directed to the corresponding author/s.

## Ethics Statement

We confirm that the ethical policies of the journal, as noted on the journal's author guidelines page, have been adhered to. This study was approved by the Animal Ethics Committee of Hunan Agricultural University, Hunan, China.

## Author Contributions

NW and JM conceived and designed the experiments. JM, DW, and YZ designed and carried out the PCV3 and PPV7 RT-PCR detection. JM, SZ, and CM conducted statistical analysis on the data. JM, AW, and NW contributed to writing and revision of the manuscript. All authors read and approved the final manuscript.

## Conflict of Interest

The authors declare that the research was conducted in the absence of any commercial or financial relationships that could be construed as a potential conflict of interest.

## Publisher's Note

All claims expressed in this article are solely those of the authors and do not necessarily represent those of their affiliated organizations, or those of the publisher, the editors and the reviewers. Any product that may be evaluated in this article, or claim that may be made by its manufacturer, is not guaranteed or endorsed by the publisher.
